# Increased Bone Marrow Interleukin-7 (IL-7)/IL-7R Levels but Reduced IL-7 Responsiveness in HIV-Positive Patients Lacking CD4+ Gain on Antiviral Therapy

**DOI:** 10.1371/journal.pone.0015663

**Published:** 2010-12-31

**Authors:** Giusi Maria Bellistrì, Anna Casabianca, Esther Merlini, Chiara Orlandi, Giulio Ferrario, Luca Meroni, Massimo Galli, Mauro Magnani, Antonella d'Arminio Monforte, Giulia Marchetti

**Affiliations:** 1 Department of Medicine, Surgery and Dentistry, Clinic of Infectious Diseases, “San Paolo” Hospital, University of Milan, Milan, Italy; 2 Department of Clinical Sciences, Chair of Infectious Diseases and Tropical Medicine, “Luigi Sacco” Hospital, University of Milan, Milan, Italy; 3 Institute of Biological Chemistry “Giorgio Fornaini”, University of Urbino, Urbino, Italy; University of Leuven, Rega Institute, Belgium

## Abstract

**Background:**

The bone marrow (BM) cytokine *milieu* might substantially affect T-lymphocyte homeostasis in HIV-positive individuals. Interleukin-7 (IL-7) is a bone marrow-derived cytokine regulating T-cell homeostasis through a CD4+-driven feedback loop. CD4+ T-lymphopenia is associated with increased free IL-7 levels and reduced IL-7R expression/function, which are only partially reverted by highly active antiretroviral therapy (HAART). We investigated the BM production, peripheral expression and signaling (pStat5+ and Bcl-2+ CD4+/CD8+ T cells) of IL-7/IL-7Rα in 30 HAART-treated HIV-positive patients who did not experience CD4+ recovery (CD4+ ≤200/µl) and who had different levels of HIV viremia; these patients included 18 immunological nonresponders (INRs; HIV-RNA≤50), 12 complete failures (CFs; HIV-RNA>1000), and 23 HIV-seronegative subjects.

**Methods:**

We studied plasma IL-7 levels, IL-7Rα+CD4+/CD8+ T-cell proportions, IL-7Rα mRNA expression in PBMCs, spontaneous IL-7 production by BM mononuclear cells (BMMCs), and IL-7 mRNA/IL-7Rα mRNA in BMMC-derived stromal cells (SCs). We also studied T-cell responsiveness to IL-7 by measuring the proportions of pStat5+ and Bcl-2+ CD4+/CD8+ T cells.

**Results:**

Compared to HIV-seronegative controls, CFs and INRs presented elevated plasma IL-7 levels and lower IL-7Rα CD4+/CD8+ cell-surface expression and peripheral blood production, confirming the most relevant IL-7/IL-7R disruption. Interestingly, BM investigation revealed a trend of higher spontaneous IL-7 production in INRs (p = .09 vs. CFs) with a nonsignificant trend toward higher IL-7-Rα mRNA levels in BMMC-derived stromal cells. However, upon IL-7 stimulation, the proportion of pStat5+CD4+ T cells did not increase in INRs despite higher constitutive levels (p = .06); INRs also displayed lower Bcl-2+CD8+ T-cell proportions than controls (p = .04).

**Conclusions:**

Despite severe CD4+ T-lymphopenia and a disrupted IL-7/IL-7R profile in the periphery, INRs display elevated BM IL-7/IL-7Rα expression but impaired T-cell responsiveness to IL-7, suggesting the activity of a central compensatory pathway targeted to replenish the CD4+ compartment, which is nevertheless inappropriate to compensate the dysfunctional signaling through IL-7 receptor.

## Introduction

Interleukin-7 (IL-7) is a type-1 stromally produced cytokine that plays a crucial role for T cell biology, enhancing thymocyte production [1], [2], “homeostatic” proliferation, survival of memory and naïve peripheral T cells [3], [4], type-1 immune responses and CD8^+^ T-cell cytotoxicity [5]. IL-7 signals through the IL-7 receptor (IL-7R) comprising an IL-7-specific-α-chain (IL-7Rα) and a common γ-chain (γ_c_) expressed on thymocytes, T lymphocytes, and bone marrow (BM) macrophages. Downstream IL-7 signaling involves Janus kinase 1 (Jak1), Jak3, Src kinases, phosphatidylinositol-3 kinase (PI3K), signal transducer and activator of transcription 3 (Stat3) and Stat5. The survival function of IL-7 is largely mediated through the maintenance of a favorable balance of anti-apoptotic Bcl-2 family members [6].

In several lymphopenic conditions, the IL-7/IL-7R axis has been shown to be crucial in sustaining peripheral T-cell homeostasis via a rise in circulating IL-7 levels that acts as a survival signal to both lymphoid progenitors and mature circulating T lymphocytes [7], [8]. In line with this model, CD4+ T-lymphopenia in the course of HIV disease is characterized by a substantial increase in IL-7 plasma levels, and yet such an IL-7-enriched *milieu* fails to preserve the peripheral T-cell pool. Aside from the immunodestructive effects of HIV-1, a major cause of the failure of IL-7 to sustain peripheral T-lymphocyte homeostasis might be the down-regulation of IL-7Rγα expression and suppression of IL-7Rα function on peripheral T lymphocytes [9], [10], [11], which might counteract the positive effect of IL-7 on T-cell homeostasis.

CD4+ T-cell reconstitution following the initiation of highly active antiretroviral therapy (HAART) is associated with a normalization of the IL-7/IL-7R axis that comprises reduced circulating IL-7 and increased IL-7Rα expression on T cells, although the levels seen in healthy HIV-seronegative individuals are not reached. However, the altered responsiveness of T cells to IL-7 has been demonstrated in HAART-treated patients, suggesting the persistence of IL-7/IL-7R dysfunction [9], [10], [11].

Despite the fact that the majority of patients undergoing HAART undergo full viro-immunologic reconstitution, up to 30% of individuals fail to experience peripheral CD4+ T-cell rescue with either suppressed or unsuppressed viremia and have an increased rate of clinical progression [12], [13], [14], [15]. Most recently, inefficient CD4+ gain on HAART has been associated with reduced recovery of T-cell responsiveness to IL-7 [16], [17]. Furthermore, patients with failed CD4+ recovery on HAART display specific alterations in the bone marrow, the primary organ contributing to IL-7 synthesis, which includes altered clonogenic capability, stromal cell dysfunction and imbalanced cytokine *milieu* [18], [19].

Collectively, these findings suggest a crucial influence of the IL-7/IL-7R axis on CD4+ T-lymphocyte reconstitution, and yet the reciprocal interactions of IL-7/IL-7R expression within lymphoid organs and peripheral blood are still poorly understood. The levels of circulating IL-7 might either reflect changes in cytokine production at IL-7-producing sites via a feedback loop with peripheral T-cell pool [1] or follow the dynamics of IL-7Rα cell-surface expression [20], [21]. Analogously, the level of IL-7Rα expression is regulated by both the free IL-7 level [11], [22] and HIV-mediated effects [23], [24].

We aimed to comprehensively investigate the production, peripheral dynamics and function of IL-7/IL-7R in HIV-positive patients failing to recover CD4+ counts following initiation of HAART. In particular, as HIV antigenemia has been shown to affect both bone marrow function and IL-7R peripheral expression [24], [25], we reasoned that HAART-treated lymphopenic patients with full viremia suppression (immunological nonresponders, INRs) might feature a different IL-7/IL-7R profile than individuals with complete immune-virological failure (complete failure, CFs).

## Results

### Patient characteristics

Thirty HIV-positive patients were recruited: 18 INRs and 12 CFs. Subjects were comparable with respect to age, sex, risk factors for HIV, AIDS diagnosis, and HAART length and regimen at the time of analysis (Table 1).

**Table 1:** Patients' characteristics.

Characteristics	INRs (n = 18)	CFs (n = 12)	P
**Age, years** *	47±8	43±9	0.278
**Sex°** ** Male** ** Female**	15 (83)3 (17)	11 (92)1 (8)	0.632
**Risk Factors°** § ** TD** ** ES** ** Other**	3/16 (19)11/16 (69)2/16 (32)	4/10 (40)4/10 (40)2/10 (20)	0.343
**Time from HIV diagnosis, years** *	5±6	8±5	0,229
**Previous AIDS diagnosis°** ** yes**	12 (67)	8 (67)	1.000
**Current CD4/µL** *	143±84	184±164	0.448
**Current CD4%** *	13±6	18±16	0.324
**Nadir CD4/µL** *	59±53	106±112	0.171
**Nadir CD4%** *	6±5	6±7	0.971
**Current HIV RNA, log_10_ cp/mL** *	1.8±0	3.9±1.3	0.0001
**HAART regimen°** ** 2NRTI+1PI** ** 2NRTI+1NNRTI** ** Other:** ** (1NRTI+1NNRTI+1PI** ** 1NRTI+1PI+raltegravir** ** 3NRTI+1PI+enfuvirtide**	14 (78)2 (11)2 (11)101	8 (67)2 (17)2 (17)110	0.844
**HAART length, months** *	29±32	57±39	0.098

**NOTE.** INRs, Immunologic Non Responders: HIV-RNA<50 cp/mL, CD4≤200/µL; CFs, Complete Failures: HIV-RNA>100 cp/mL, CD4≤200/µL.

*Data are mean ± standard deviation;

° data are *n* (%).

§ Risk factor for HIV infection was available in 16 out of 18 INRs and in 10 out of 12 CFs. HAART, Highly Active Antiretroviral Therapy. NRTI, nucleoside reverse-transcriptase inhibitor; NNRTI, nonnucleoside reverse-transcriptase inhibitor; PI, protease inhibitor.

As per inclusion criteria, CFs displayed significantly higher mean HIV RNA levels (p = .0001) and comparable mean absolute and percent current CD4+ counts (p = .4) and nadir (p = .2) (Table 1). No differences were shown with respect to HAART regimen and duration between the groups; 83% of the patients (25/30) were on a protease inhibitor (PI)-based regimen (Table 1).

### INRs had high circulating IL-7 levels and low IL-7Rα expression on T cells

**Figure 1:**
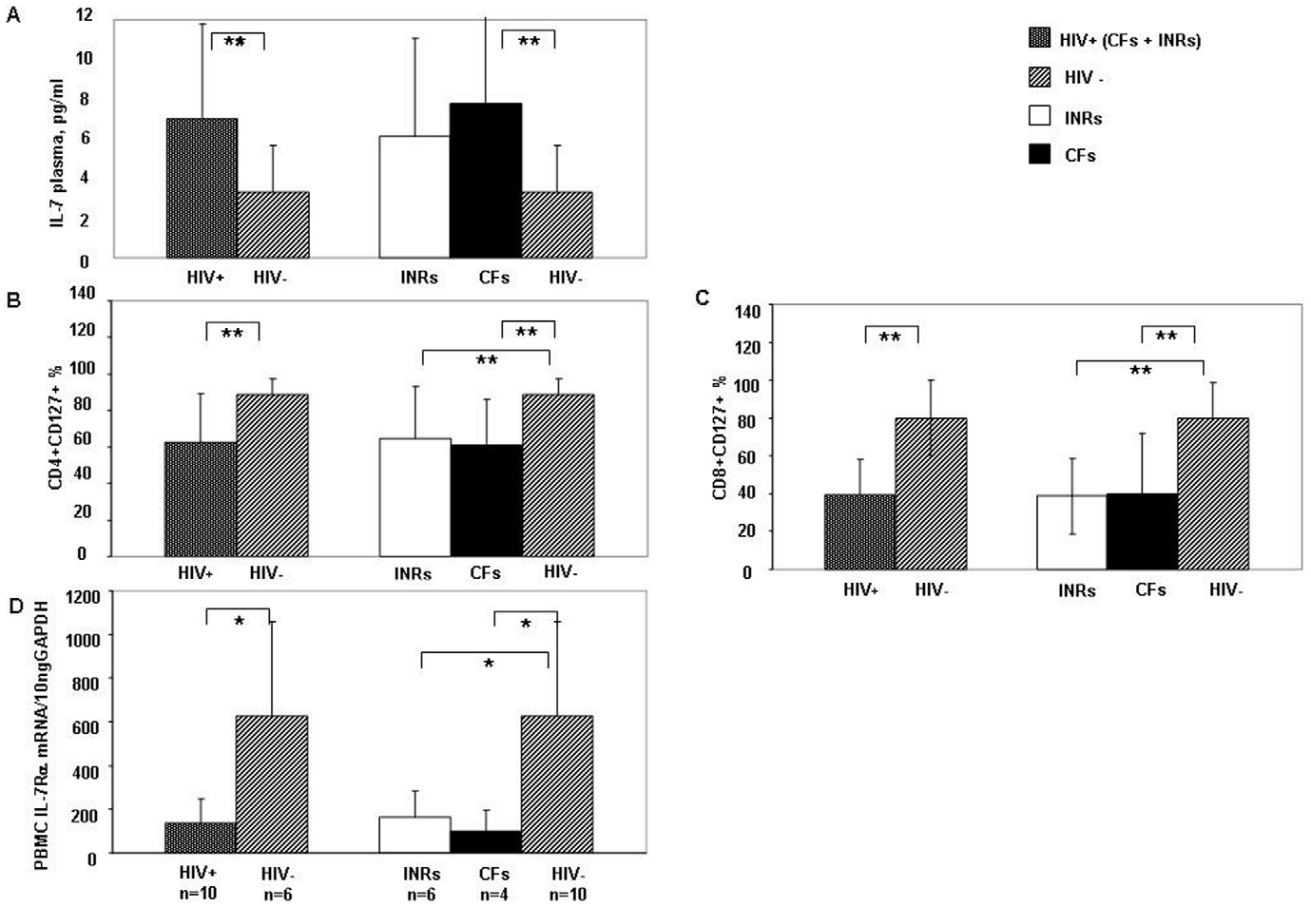
IL-7 plasma levels and IL-7Rα expression and production in peripheral blood. Levels of plasma IL-7 (A), IL-7Rα expression on CD4+ and CD8+ T cells (B–C), and IL-7Rα production in peripheral blood (D) differ between HIV-positive HAART-treated subjects and HIV-seronegative subjects. HIV-positive subjects were subdivided into two groups: subjects with low CD4+ counts and full suppression of viral replication (immunological nonresponders, INRs) or patients with low CD4+ T-cell counts and elevated viral replication (complete failures, CFs). Compared to HIV-seronegative controls, both INRs and CFs displayed augmented IL-7 plasma levels (A) and reduced proportions of IL-7Rα+ CD4+ and IL-7Rα+ CD8+ T cells (B and C). IL-7Rα production was measured in the peripheral blood of 10 unselected HIV-positive patients (6 INRs and 4 CFs) and 6 HIV-seronegative subjects. Both INRs and CFs had significantly lower IL-7Rα mRNA levels than HIV-seronegative controls (D). Histogram heights and error bars denote the mean and standard deviation, respectively, of the indicated parameters. ^*^ *P*<.05; ^**^ *P*<.01.

Compared to HIV-seronegative subjects, HIV-positive patients had significantly higher mean IL-7 plasma levels (HIV positive: 7±4.7 pg/ml, HIV seronegative: 3.3±2.4 pg/ml, p = .002) (Figure 1A), with elevated circulating IL-7 levels in both INRs and CFs, reaching significance for CFs (INRs: 6.3±5.1 pg/ml; CFs: 7.5±4.6 pg/ml; p = .7 for INRs vs. CFs; p = .1 for INRs vs. HIV-seronegative subjects; p = .003 for CFs vs. HIV-seronegative subjects) (Figure 1A).

As for IL-7Rα expression, HIV-positive patients had significant lower mean CD4+/CD8+ CD127+ T-cell proportions (CD4+CD127+, HIV positive: 62.7±26.4%, HIV seronegative: 88.6±8.4%, p = .0001; CD8+CD127+, HIV positive: 39.4±25.1%, HIV seronegative: 80±18.6%; p = .0001; Figure 1B–C). INRs displayed significantly lower levels of CD4+CD127+ T cells than HIV-seronegative subjects and levels comparable to those of CFs (INRs: 63.1±29.1%; CFs: 62.4±24.7; p = .99 for INRs vs. CFs; p = .02 for INRs vs. HIV-seronegative subjects; p = .002 for CFs vs. HIV-seronegative subjects) (Figure 1B). A similar trend was shown for CD8+ IL-7Rα expression (INRs: 38.8±14.2%; CFs: 39.9±32.2%; p = .99 for INRs vs. CFs; p = .0001 for INRs vs. HIV-seronegative subjects, p = .001 for CFs vs. HIV-seronegative subjects) (Figure 1C).

### INRs had reduced IL-7Rα expression by PBMCs

According to cell-surface expression, HIV-positive patients presented significantly lower IL-7Rα mRNA levels than HIV-seronegative subjects (HIV positive: 138.7±109.9; HIV seronegative: 625.3±434.8 ng IL-7Rα/10 ng GAPDH; p = .03) (Figure 1D). Furthermore, both INRs and CFs had significantly lower PBMC IL-7Rα mRNA levels vs. HIV-seronegative controls (INRs: 163.3±120.2 ng IL-7 Rα/10 ng GAPDH; CFs: 101.7±95.7 ng IL-7 Rα/10 ng GAPDH; p = .6 for INRs vs. CFs; p = .04 for INRs vs. HIV-seronegative subjects; p = .03 for CFs vs. HIV-seronegative subjects) (Figure 1D).

### INRs displayed augmented IL-7/IL-7Rα expression in bone marrow mononuclear cell (BMMC) cultures

**Figure 2:**
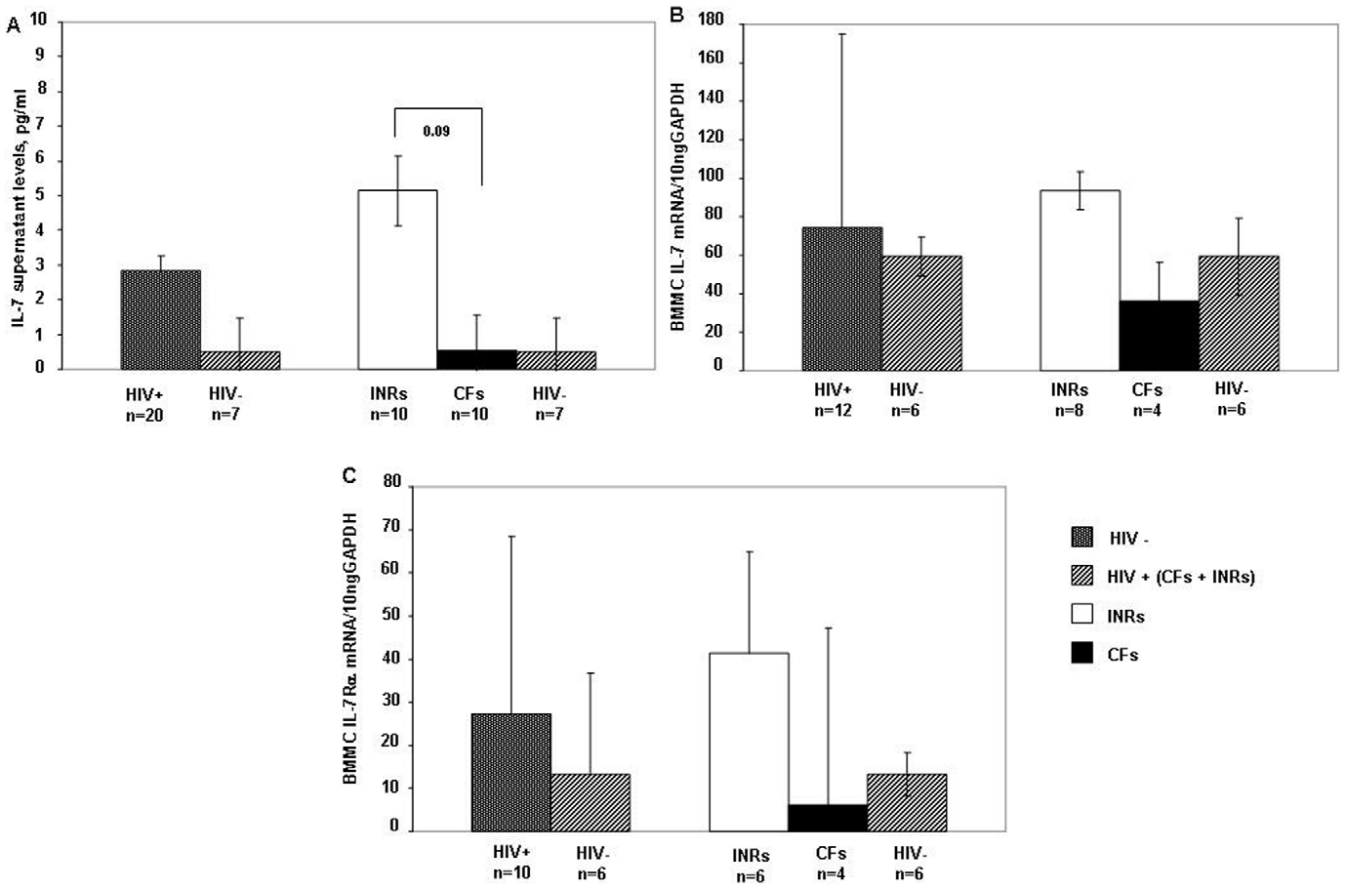
IL-7/IL-7Rα production by bone marrow stromal cells. IL-7 production was analyzed by (i) measuring IL-7 levels in the supernatants of bone marrow mononuclear cells (BMMCs) from 20 unselected HIV-positive (10 INRs, 10 CFs) and 7 HIV-seronegative patients (A); and (ii) measuring IL-7 mRNA levels in BMMC-derived stromal cells from 12 unselected HIV-positive (8 INRs, 4 CFs) and 6 HIV-seronegative patients (B). IL-7Rα bone marrow expression was measured in 10 unselected (6 INRs, 4 CFs) and 6 HIV-seronegative patients (C). IL-7/IL-7Rα bone marrow production in BMMCs differed between HIV-positive HAART-treated subjects and HIV-seronegative subjects. HIV-positive subjects were subdivided into two groups: subjects with low CD4+ counts and full suppression of viral replication (immunological nonresponders, INRs) and patients with low CD4+ T-cell counts and elevated viral replication (complete failures, CFs). HIV-positive patients presented increased bone marrow production of IL-7 (A–B) and IL-7Rα (C). Separate analyses of HIV-positive patients with different viro-immunological responses to HAART revealed a tendency for higher IL-7/IL-7Rα production in INRs compared to other patients. Histogram heights and error bars denote the mean and standard deviation, respectively, of the indicated parameters.

Having shown differences in IL-7/IL-7Rα expression and production in peripheral blood, we investigated the expression at the bone marrow level. According to data for the periphery, HIV-positive patients as a whole presented a trend toward higher IL-7 levels in BMMC supernatants compared to HIV-seronegative subjects, though the difference was not statistically significant (HIV positive: 2.8±7.7 pg/ml, HIV seronegative: 0.5±0.4 pg/ml; p = .7) (Figure 2A). Interestingly, following the division patient into groups, INRs showed the highest level of spontaneous BMMC IL-7 production (INRs: 5.1±10.7 pg/ml, CFs: 0.5±0.8 pg/ml; p = .09 for INRs vs. CFs; p = .4 for INRs vs. HIV-seronegative subjects; p = 1 for CFs vs. HIV-seronegative subjects) (Figure 2A).

Real-time PCR analysis of BMMC-derived SCs revealed comparable IL-7 mRNA levels between HIV-positive patients and HIV-seronegative subjects (HIV positive: 74.5±100.7 ng IL-7/10 ng GAPDH; HIV seronegative: 59.3±113.3 ng IL-7/10 ng GAPDH; p = .4). Interestingly, INRs had a higher mean IL-7 mRNA level than CFs and HIV-seronegative subjects, but this difference was not statistically significant (INRs: 93.6±118.2 ng of IL-7/10 ng GAPDH, CFs: 36.2±41 ng of IL-7/10 ng GAPDH; p = .5 for CFs vs. INRs; p = .8 for INRs vs. HIV-seronegative subjects; p = .9 for CFs vs. HIV-seronegative subjects) (Figure 2B).

Furthermore, BMMC-derived SCs were investigated for IL-7Rα expression by mRNA quantization. HIV-positive patients had higher IL-7Rα mRNA levels than HIV-seronegative controls, although this difference was not statistically significant (HIV positive: 27.2±41.27 IL-7Rα/10 ng GAPDH, HIV seronegative: 13.17±23.57 ng IL-7Rα/10 ng GAPDH; p = .2). Interestingly, INRs tended to have higher IL-7Rα mRNA levels than CF patients and HIV-seronegative controls, although these difference were not statistically significant (INRs: 41.33±49.64 ng IL-7 Rα/10 ng GAPDH, CFs: 6±2.16 ng IL-7 Rα/10 ng GAPDH; p = .3 for INRs vs. CFs; p = .5 for INRs vs. HIV-seronegative subjects; p = .7 for CFs vs. HIV-seronegative subjects) (Figure 2C).

### INRs had diminished levels of pStat5+CD4+ and Bcl-2+CD8+ T cells following IL-7 stimulation

Given the higher IL-7 BM production despite low CD4+ counts in INRs, we investigated the *ex vivo* response to IL-7.

**Figure 3:**
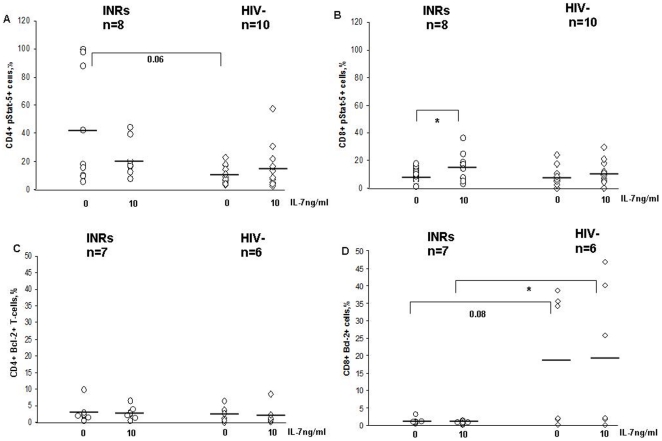
IL-7 signaling in CD4+ and CD8+ T-cell subsets. IL-7 responsiveness was analyzed by measuring the constitutive and IL-7-induced levels of phosphorylated signal transducer and activator of transcription 5 (pStat5) in CD4+ and CD8+ T cells from 8 unselected INRs compared to 10 HIV-seronegative controls (A and B). INRs displayed a trend of higher constitutive percentages of CD4+pStat5+ T cells vs. HIV-seronegative controls. Following IL-7 stimulation, the proportion of CD4+pStat5+ T cells tended to decrease in INRs (A), whereas no major changes were observed for CD8+pStat5+ T cells (B). Constitutive and IL-7-induced expression levels of the anti-apoptotic molecule Bcl-2 were comparatively measured in seven unselected INRs and six HIV-seronegative controls (C and D). No major differences in the proportions of CD4+Bcl-2+ T cells were observed between INRs and HIV-seronegative controls (C), whereas INRs displayed significantly lower proportions of constitutive and IL-7-induced CD8+Bcl-2+ T cells (D). ^*^ *P*<.05.

Compared to HIV-seronegative subjects, INRs had higher constitutive pStat5+CD4+ levels, whereas no differences were shown between constitutive pStat5+CD8+ T cell levels (pStat5+CD4+, INRs: 42.9±43.5%, HIV-seronegative subjects: 10.7±6.8%, p = .06; pStat5+CD8+, INRs: 10.4±5.2%, HIV-seronegative subjects: 8.4±7.1%, p = .3) (Figure 3A–B). Interestingly, in INRs, IL-7 stimulation resulted in a nonsignificant reduction in the proportion of pStat5+CD4+ T cells (20.2±13.8%; p = .2); the proportion of pStat5+CD4+ T cells tended to increase in HIV-seronegative controls (16.6±16.8%; p = .2) (Figure 3A). Conversely, the proportion of pStat5+CD8+ T cells tended to increase upon IL-7 stimulation in both patient groups, reaching significance in INRs (INRs: 15.1±10.7%, p = .04; HIV-seronegative subjects: 11.2±8.6%, p = .2) (Figure 3B).

No major differences were shown with respect to the proportion of constitutive and IL-7-stimulated Bcl-2+CD4+ T cells (INRs: 2.9±3.22%, HIV-seronegative subjects: 2.6±2.5%, p = .9; IL-7-mediated Bcl-2+CD4+, INRs: 2.6±2%, HIV-seronegative subjects: 2.3±3.2%, p = .4) (Figure 3C). Conversely, compared to HIV-seronegative subjects, INRs had lower constitutive and IL-7-stimulated Bcl-2+CD8+ T-cell proportions (basal Bcl-2+CD8+ T cells, INRs: 1±0.9%, HIV-seronegative subjects: 18.7±19.1%, p = .08; IL-7-induced Bcl-2+CD8+ T cells INRs: 0.7±0.6%, HIV-seronegative subjects: 19.4±21%, p = .04) (Figure 3D). No major changes in the proportions of Bcl-2+ and CD8+ T cells were observed following IL-7 stimulation in both patient groups (p = .2 and p = .9 for INRs and HIV-seronegative subjects, respectively) (Figure 3D).

## Discussion

The failure to gain CD4+ T cells and the time spent with nonprotective CD4+ counts following HAART initiation are associated with increased morbidity and mortality [26], [27], making the identification of alternative therapeutic approaches to promote immune reconstitution essential. Recently, adjuvant recombinant human IL-7 (rhIL-7) was demonstrated to be effective in sustaining CD4+ recovery in HIV-positive patients with different degrees of immune deficiency [28], [29]. Theoretically, due to its role as a major regulator of T-lymphocyte homeostasis, IL-7 might be particularly helpful in patients on HAART with persistent CD4+ lymphopenia, but the actual clinical value of IL-7 treatment in these patients is questioned by the evidence of already elevated endogenous IL-7 levels and reduced IL-7Rα expression [16], [17], [28], [29]. Therefore, we studied the bone marrow expression and peripheral levels of IL-7 and IL-7Rα in HIV-positive patients failing to recover CD4+ counts following initiation of HAART with and without ongoing HIV viral replication.

In addition to confirming the elevated plasma IL-7 levels and reduced IL-7Rα expression in circulating T cells in HIV-positive individuals [10], [20], [30], [31], we found that HIV-positive patients had levels of bone marrow IL-7 production comparable to that of healthy HIV-seronegative controls, with a trend for higher IL-7Rα bone marrow expression compared to HIV-seronegative individuals. As these findings were unexpected given the altered bone marrow architecture and function resulting from HIV infection [18], [32], [33], [34], [35], we separately analyzed HIV-positive individuals to test the hypothesis that the bone marrow functions of subjects differed according to the immune-virological response to HAART.

Despite a similar peripheral profile characterized by severe CD4+ T-lymphopenia, heightened plasma IL-7 levels and reduced IL-7Rα+CD4+/CD8+ T cell levels, the investigation of the bone marrow compartment revealed divergent IL-7/IL-7R production between INRs and CFs.

Patients with complete viro-immunological failure to HAART displayed totally hampered production of IL-7 and IL-7Rα, likely due to the complete loss of function of both bone marrow stromal cells and peripheral lymphocytes. This finding is in line with evidence suggesting that during the course of HIV infection, bone marrow stromal auxiliary cells are persistently infected and dysfunctional, impairing the marrow's hematopoietic functions [18], [35], [36]. Thus, elevated levels of circulating IL-7 in CFs likely result from release by down-regulated cell-surface IL-7Rα levels and not from increased production.

Our findings indicate the activity of opposing pathways in INRs, in which stromal cell production of both IL-7 and IL-7Rα seems to be elevated. Newly produced IL-7 might be released into the peripheral blood and rapidly bind to cell-surface receptors, resulting in the down-regulation of IL-7R in both CD4+ and CD8+ T cells [37].

With this perspective, equally reduced peripheral IL-7Rα production and expression reflect dissimilar immune pathways. In CFs, reduced peripheral IL-7Rα production and expression might be due to IL-7Rα down-regulation driven by both elevated circulating IL-7 levels, a direct viral effect [24], [25], and HIV-induced immune activation [22], [23], whereas in INRs, reduced peripheral IL-7Rα production and expression might result from down-regulation of IL-7 receptor expression on CD4+ and CD8+ T cells following IL-7/IL-7R engagement [37].

Aiming to reconcile increased IL-7/IL-7R production with persistent CD4+ T-lymphopenia in spite of removing the viral challenge in INRs, we investigated the effect of reduced IL-7-mediated signaling on T-lymphocyte homeostasis.

When compared to HIV-seronegative subjects, INRs displayed higher constitutive pStat5 levels exclusively within CD4+ cells, suggesting the persistence of HIV-induced up-regulation of Stat-dependent signaling pathways despite viremia suppression [38], [39]. However, IL-7 stimulation did not result in increased pStat5+ T-cell proportions, confirming the reduced IL-7 responsiveness of the peripheral T cells [16], [17].

Our data suggest a model whereby CD4+ breakdown in the absence of viral challenge is associated with compensatory pathway(s) at the level of bone marrow stromal cells, resulting in increased IL-7 production and activated Stat5 signaling, specifically in CD4+ T cells. It is tempting to speculate that this is a compensatory pathway targeted to maintain peripheral T-lymphocyte homeostasis. However, this supposition may be inappropriate for continuous CD4+ exhaustion given the failure of the IL-7-mediated peripheral signaling. One possibility is that with the presence of such compensatory constitutive bone marrow activation, the IL-7/IL-7R system might experience a “functional exhaustion” with respect to the continuous demand provided by peripheral T-lymphopenia, resulting in an overall desensitization to further IL-7 stimulation.

Further studies on the regulatory pathways behind bone marrow IL-7/IL-7R production and IL-7Rα-mediated signaling in HIV/AIDS patients are needed to identify the most efficacious clinical use of IL-7 in the (adjuvant) treatment of HIV-positive patients.

## Methods

### Patients

Between October 2006 and June 2009, we performed a cross-sectional study including 30 HIV-positive patients who were followed at the Clinics of Infectious Diseases, “Luigi Sacco” and “San Paolo” Hospitals, University of Milan. Inclusion criteria were stable HAART for at least six months and CD4+ ≤200/µL over the last six months. Based upon virological response to six-month HAART, patients were divided into two groups: INRs (HIV-RNA≤50 cp/ml) and CFs (HIV-RNA>1000 cp/ml).

As controls, we included 23 HIV-seronegative patients undergoing heart surgery.

All enrolled patients provided written informed consent according to the Ethics Committees of our Institutions (Comitato Etico, Ospedale “San Paolo” and Comitato Etico, Ospedale “Luigi Sacco”, Milan, Italy). Both ethics committees specifically approved this study.

### IL-7 plasma concentration

IL-7 plasma concentrations were evaluated by ELISA (Quantikine HS human IL-7; R&D, Milan, Italy) according to manufacturer's instructions.

### Proportion of IL-7Rα+CD4+ and CD8+ T cells

The proportions of IL-7Rα (CD127)+ CD4+ T cells and CD8+ T cells were evaluated by flow cytometry (Coulter ESP; Beckman Coulter, Hialeah, FL, USA). Briefly, 50 µl of fresh whole peripheral blood was stained with fluorescently labeled antibodies (fluorescein isothiocyanate [FITC], phycoerythrin [PE], and phycoerythrin-cyanine 5 [PCy5]) (CD4+-FITC, Becton Dickinson, San Jose, CA, USA; CD127+-PE and CD8+-PCy5, Coulter, FL, USA). The following combination of monoclonal antibodies was used: CD4+/CD8+/CD127+.

### Cultures of bone marrow mononuclear cells (BMMCs)

Bone marrow aspirates were collected in EDTA. Ficoll-separated BMMCs were cultured in 12-well plates in IMDM medium containing 20% FCS, 100 UI/ml L-glutamine and 100 UI/ml penicillin-streptomycin at a concentration of 1×10^6^ cells/ml in a total volume of 3 ml/well.

### Spontaneous IL-7 production by cultured BMMCs

BMMC cultures were incubated at 37°C in humidified air with 5% CO_2_ in the absence of stimuli to verify spontaneous production of IL-7. Briefly, 24 hours after BMMC confluence, supernatants were collected and IL-7 levels were measured by ELISA (Quantikine HS human IL-7; R&D, Milan, Italy) according to the manufacturer's instructions.

### IL-7 mRNA quantification from BMMC-derived stromal cells (SCs)

Following BMMC culture as described above, nonadherent cells were removed from cultures at specific intervals and were replaced by 1000 µl of fresh supplemented IMDM. The cultures were subsequently maintained until stromal confluence (3-4 weeks). BMMC-derived SCs were collected by trypsinization and were used to quantify IL-7 mRNA by real-time PCR. (**i) RNA extraction and reverse transcription**. Total RNA was isolated from BMMC-derived SCs (TRIzol®, Invitrogen, Carlsbad, CA, USA). Potential genomic DNA contamination was removed by incubation with RNase-free DNase I (Invitrogen, Carlsbad CA, USA). From each sample, 4 to 8 µl of total RNA (corresponding to about 250–500 ng) was used for cDNA synthesis using the SuperScript™ III First-Strand Synthesis System for RT-PCR according to the manufacturer's instructions (Invitrogen, Carlsbad CA, USA). **(ii) IL-7 mRNA quantification**. A portion of the cDNA mixture, corresponding to 1/3, 1/6, 1/21, or 1/210, was subjected to SYBR green-based IL-7 and GAPDH mRNA real-time PCR [40]. All amplifications were carried out on a 7500 Real-Time PCR System (Applied Biosystems, Foster City, CA, USA) in a final volume of 50 µl with 100 nM of primers using the Hot-Rescue Real-Time PCR Kit-SG (Diatheva s.r.l., Fano, Italy). For IL-7 amplification, after one cycle at 95°C for 10 min, a two-step PCR procedure was used consisting of 15 sec at 95°C and 45 sec at 68°C for 45 cycles. For GAPDH amplification, the cycling conditions were 10 min at 95°C followed by 45 cycles of 15 sec at 95°C, 15 sec at 60°C and 35 sec at 72°C. To verify the specificity of the PCR products, a dissociation curve analysis was performed. He IL-7 and GAPDH primers amplified 157-bp and 176-bp cDNA fragments, respectively [41]. An external RNA standard curve was used to quantify IL-7 mRNA levels. Ten-fold serial dilutions from 10^7^ to 10^3^ of synthetic IL-7 RNA molecules synthesized using the T7 RiboMAX™ Express Large-Scale RNA Production System (Promega, Milan, Italy) were reverse transcribed and amplified in the same run with unknown samples. For data normalization, variable amounts (500-100-50-10-5 ng) of mRNA (extracted from the PBMCs of healthy donors) were used to generate a “gold standard” for GAPDH quantification. The standard curves were created automatically by the Applied Biosystems software based upon threshold (Ct) values. A curve was accepted when the slope was between -3.50 and -3.32 (93–100% efficiency) and when the minimum value of the correlation coefficient (R^2^) was 0.98. Data were expressed as IL-7 mRNA copy number/10 ng GAPDH mRNA.

### IL-7Rα mRNA quantification from PBMCs and BMMC-derived SCs

Following RNA extraction/reverse transcription as described above, detection of IL-7Rα mRNA in PBMCs and BMMC-derived SCs was performed using the Assay on Demand Kit (ID: Hs00233682_m1 Applied Biosystems). The “gold standard” described above was used for IL-7Rα and GAPDH mRNA quantification. Data were expressed as ng IL-7Rα mRNA/10 ng GAPDH mRNA.

### Assessment of Stat5 phosphorylation

Ficoll-separated PBMCs were stained with fluorescently labeled antibodies to cell surface markers (CD4+-FITC, CD8+-FITC, Becton Dickinson, San Jose, CA, USA). PBMCs (2×10^6^ cells) were incubated in medium (serum-free RPMI) with or without IL-7 (10 ng/mL) for 15 min at 37°C, washed, treated with FACS-lysing solution (Becton Dickinson, San Jose, CA, USA), and stained with anti-pStat5 perCP-Cy5.5 (BD Biosciences, San Jose, CA, USA) for 1 hour at RT in the dark. The following combinations of monoclonal antibodies were used: CD4+/pStat5 and CD8+/pStat5.

### Assessment of Bcl-2

Ficoll-separated PBMCs were stained with antibodies to cell surface markers (CD4+-PE, CD8+-PE, Becton Dickinson, San Jose, CA, USA). PBMCs (4×10^6^ cells) were incubated in medium (serum-free RPMI) with or without IL-7 (10 ng/mL) for 24 hours at 37°C, washed, treated by FACS-lysing solution (Becton Dickinson, San Jose, CA, USA), and stained with anti-Bcl-2-FITC (BD Biosciences, San Jose, CA, USA). The following combinations of monoclonal antibodies were used: CD4+/Bcl-2 and CD8+/Bcl-2. pStat5/Bcl-2 analysis was conducted using a BD FACSort (BD Biosciences, San Jose, CA, USA).

### Statistics

Baseline categorical and continuous parameters were compared using Fisher's exact test, the Pearson chi-square test and Student's *t* test for independent samples, respectively. Variables for HIV-positive patients and healthy controls were compared with Student's *t* test and the Mann-Whitney nonparametric U test. The same variables were also compared between HIV-positive subjects, INRs and CFs using one-way ANOVA followed by the Games-Howell *post hoc* test for multiple comparisons. For comparisons between the parameters of stimulated and unstimulated cells, the Wilcoxon matched-pairs signed rank test was used. Statistics were calculated using SPSS software (version 18.0).
